# Zinc Supplementation Trial in Pediatric Chronic Kidney Disease: Effects on Circulating FGF-23 and Klotho

**DOI:** 10.1177/20543581241234723

**Published:** 2024-03-13

**Authors:** V. Belostotsky, S. A. Atkinson, G. Filler

**Affiliations:** 1 Division of Nephrology, Department of Paediatrics, McMaster Children’s Hospital, Hamilton, ON, Canada; 2 Department of Paediatrics, McMaster University, Hamilton, ON, Canada; 3 Division of Nephrology, Departments of Paediatrics and Medicine, Western University, London, ON, Canada; 4 Lilibeth Caberto Kidney Clinical Research Unit, London, ON, Canada

**Keywords:** chronic kidney disease, zinc, FGF-23, Klotho, clinical trial, children

## Abstract

**Background:**

Zinc status, its role in bone metabolism and efficacy of deficiency correction has not been well studied in children with chronic kidney disease (CKD).

**Objectives:**

The primary objective was to investigate whether 3 months of oral zinc supplementation corrects zinc deficiency in children with CKD who have native or transplanted kidneys. The secondary objective was to compare circulating intact FGF-23 (iFGF-23), c-terminal FGF-23 (cFGF-23), and Klotho between zinc-sufficient and zinc-deficient children with CKD and to assess the relationship between circulating zinc, iFGF-23, cFGF-23, Klotho, bone biomarkers, copper, and phosphate excretion pre-supplementation and post-supplementation of zinc.

**Methods:**

Forty-one children (25 male and 16 female, age 12.94 ± 4.13 years) with CKD in native or transplanted kidneys were recruited through 2 pediatric nephrology divisions in Ontario, Canada. Of those, 14 patients (9 native CKD, 5 transplant CKD) with identified zinc deficiency (64% enrollment rate) received zinc citrate supplement for 3 months: 10 mg orally once (4-8 years) or twice (9-18 years) daily.

**Results:**

Zinc deficiency (plasma concentration < 11.5 µmol/L) was found in 22 patients (53.7%). A linear regression model suggested that zinc concentration reduced by 0.026 µmol/L (*P* = .04) for every 1-unit of estimated glomerular filtration rate (eGFR) drop. Zinc deficiency status was associated with higher serum iFGF-23; however, this was predominantly determined by the falling GFR. Zinc deficient and sufficient children had similar circulating c-FGF-23 and alpha-Klotho. Normalization of plasma zinc concentration was achieved in 8 (5 native CKD and 3 transplant CKD) out of 14 treated patients rising from 10.04 ± 1.42 to 12.29 ± 3.77 μmol/L (*P* = .0038). There were no significant changes in other biochemical measures in all treated patients. A statistically significant (*P* = .0078) rise in c-FGF-23 was observed only in a subgroup of 11 children treated with zinc but not receiving calcitriol.

**Conclusions:**

Zinc status is related to kidney function and possibly connected to bone metabolism in patients with CKD. However, it plays a minor role in fine-tuning various metabolic processes. In this exploratory non-randomized study, 3 months supplementation with zinc corrected deficiency in just over half of patients and only modestly affected bone metabolism in asymptomatic CKD patients.

## Abrégé

**Contexte:**

Le statut du zinc, son rôle dans le métabolisme osseux et l’efficacité de la correction d’une carence n’ont pas encore été bien étudiés chez les enfants atteints d’insuffisance rénale chronique (IRC).

**Objectifs:**

L’objectif principal était d’examiner si une supplémentation orale en zinc pendant trois mois pouvait corriger une carence chez les enfants atteints d’IRC avec des reins natifs ou transplantés. Les objectifs secondaires étaient de comparer les taux circulants de FGF-23 intact (iFGF-23), de FGF-23 c-terminal (cFGF-23) et de Klotho d’enfants atteints d’IRC et ayant des niveaux corrects ou déficients en zinc, puis d’évaluer la relation entre le zinc circulant, l’iFGF-23, le cFGF-23, le Klotho, les biomarqueurs osseux et l’excrétion de cuivre et de phosphate avant et après la supplémentation en zinc.

**Méthodologie:**

Un total de 41 enfants (25 garçons et 16 filles, âgés de 12,94 ±4,13 ans) atteints d’IRC avec reins natifs ou transplantés ont été recrutés par deux divisions de néphrologie pédiatrique en Ontario, au Canada. De ceux-ci, 14 patients (9 avec reins natifs; 5 avec reins transplantés) qui présentaient une carence en zinc (taux d’inclusion de 64 %) ont reçu un supplément de citrate de zinc pendant trois mois à raison de 10 mg par voie orale une fois (4 à 8 ans) ou deux fois (9 à 18 ans) par jour.

**Résultats:**

Une carence en zinc (concentration plasmatique < 11,5 µmol/L) a été constatée chez 22 patients (53,7 %). Un modèle de régression linéaire a suggéré que la concentration de zinc diminue de 0,026 µmol/L (*P* = 0,04) pour chaque chute d’une unité de DFGe. Le statut de carence en zinc était associé à un taux sérique d’iFGF-23 plus élevé; cependant, cela était principalement déterminé par la baisse du DFG. Tous les enfants, avec ou sans carence en zinc, avaient des taux circulants similaires de c-FGF-23 et d’alpha-Klotho. La normalisation de la concentration plasmatique de zinc a été obtenue chez 8 patients (5 avec reins natifs; 3 avec reins transplantés) sur les 14 qui ont été traités, passant de 10,04 ±1,42 à 12,29 ±3,77 μmol/L (*P* = 0,003 8). Aucun changement significatif n’a été observé dans les autres mesures biochimiques chez les patients traités. Une augmentation statistiquement significative (*P* = 0,007 8) du taux de c-FGF-23 a été observée uniquement dans un sous-groupe de 11 enfants traités avec du zinc, mais ne recevant pas de calcitriol.

**Conclusion:**

Le statut du zinc est lié à la fonction rénale et peut être lié au métabolisme osseux chez les patients atteints d’IRC. Il joue toutefois un rôle mineur dans le réglage fin de divers processus métaboliques. Dans cet essai exploratoire non randomisé, une supplémentation en zinc pendant trois mois a permis de corriger la carence chez un peu plus de la moitié des patients et n’a eu qu’un effet modeste sur le métabolisme osseux chez les patients asymptomatiques atteints d’IRC.

## Introduction

Children with chronic kidney disease (CKD) develop significant bone disease and cardiovascular morbidity later in life due to the accumulation of uremic toxins and phosphate.^1^ During childhood, CKD can be associated with subclinical zinc deficiency, which contributes to uremic symptoms such as anorexia, dysgeusia, parosmia, and impaired immune function.^2^ Whether such zinc deficiency contributes to the later bone and cardiovascular disease in unknown and no evidence of clinical trials exploring the efficacy of zinc supplementation on restoring serum levels to sufficient values in pediatric CKD patients was identified.

Little is known about zinc’s role in the abnormal bone metabolism and phosphate accumulation commonly observed in CKD. Fibroblast Growth Factor 23 (FGF-23) promotes phosphate clearance and becomes elevated in response to hyperphosphatemia from the early stages of CKD.^3,4^ To exert its function, FGF-23 requires the presence of the Klotho protein that circulates in blood and is also expressed by the tubular cells. Patients with CKD are unable to remove excess phosphate despite elevated FGF-23 due to low Klotho levels and at the same time FGF-23 loses its hormonal suppression effect due to the reduced expression of parathyroid Klotho.^5^ Fibroblast Growth Factor 23 accumulation has been shown to be a predictor of all-cause mortality in adult patients with advanced CKD.^6,7^ Traditional treatment for hyperphosphatemia is challenging due to difficulty with adherence to diet and to phosphorus binders use owing to side effects of poor taste and abdominal discomfort.^8^ Animal models suggest that zinc supplementation could stimulate Klotho production^9,10^; however, this approach has not been studied in humans.

The primary objective of this exploratory study was to investigate whether 3 months of oral zinc supplementation corrects zinc deficiency in children with CKD who have native or transplanted kidneys. The secondary objective was to compare intact circulating FGF-23 (iFGF-23), c-terminal FGF-23 (cFGF-23), and Klotho between zinc-sufficient and zinc-deficient children with CKD and to assess the relationship between circulating zinc, iFGF-23, cFGF-23, Klotho, bone biomarkers, copper, and phosphate excretion pre-supplementation and post-supplementation of zinc. We hypothesized that similar to animal data, correction of zinc deficiency would result in increased Klotho^9,10^ and possibly enhanced phosphate excretion.

## Methods

### Study Population

Children with CKD (native or renal transplant function) were recruited through the pediatric nephrology divisions at McMaster Children’s Hospital in Hamilton, Ontario and Children’s Hospital in London, Ontario. Patients and their parents were informed about the study by clinic nurses during their routine CKD clinic visit. Research ethics approval was obtained from Research Ethics Boards from Hamilton Health Sciences at McMaster University in Hamilton and Health Sciences at Western University in London and adhered to the Declaration of Helsinki. The trial was registered on clinicaltrials.org site (NCT02126293) and Clinical Trial Application (CTA) approval for the study was granted by Health Canada. Written informed consent was obtained by research assistants from the caregivers in every case, and—where appropriate—written assent from the minor.

Inclusion criteria included children between 4 and 18 years of age; diagnosis of CKD stage 2-4; and renal transplant recipient with declining estimated glomerular filtration rate (eGFR) of <90 mL/min/1.73 m^2^. Exclusion criteria included lack of caregiver signed consent, children younger than 4 years of age, kidney transplant recipients with eGFR>90 ml/min/1.73 m^2^; patients with vitamin D deficiency as defined by Health Canada criteria (ie, <50 nmol/L); or children with gastrointestinal disorders.

At the completion of 3 months of zinc supplementation, the same participant data, blood, and urine samples were collected and analyzed.

### Intervention

Zinc-deficient patients were prescribed zinc citrate (Zinc Lozenges, manufactured by Douglas Laboratories Inc, London, ON, Health Canada Natural Product Number [NPN] 80032476) for 3 months. As mandated by Health Canada, dosage was guided by the NPN license with 10 mg (1 lozenge) orally once a day for children aged 4 to 8 years, and 10 mg twice a day for children aged 9 to 18 years. Age difference in zinc dose is based on simplified weight adjusted dosing. Patients were advised to take zinc lozenges with meals, to minimize gastrointestinal side effects and 2-3 hours before or after taking other medications. Research assistants contacted families at 2 weeks and 2 months after the start of supplementation to ensure treatment adherence, minimize attrition and collect information on adverse events, and changes in concomitant medication or health status. Given the exploratory nature of this study, each participant’s involvement concluded after a 3-month period, as the primary objective was not to achieve complete repletion of zinc stores.

### Safety Assessment

Adult-focused literature reports that an unsafe amount of zinc absorption is >100 to 300 mg of zinc per day,^11^ which is significantly more than we used in study participants. A recent meta-analysis showed that an overall mean (95% confidence interval [CI]) urinary and endogenous fecal zinc excretion of 17.48 µg/kg/d (11.80-23.15; I^2^ = 94%) and 0.07 mg/kg/d (0.06-0.08; I^2^ = 82%), respectively, with a mean fractional zinc absorption of 26.75% (23.69-29.81; I^2^ = 99%) indicated primarily bowel excretion of zinc.^12^ Thus, the safety of zinc supplementation in CKD children should not be significantly different from other children without kidney disease. Upper tolerable levels of daily zinc intake likely to pose no risk of adverse health effects in almost all individuals are 12 mg for 4- to 8-year-olds, 23 mg for 9- to 13-year-olds and 34 for 14- to 18-year-olds.^13^ The recommended dose of zinc for Wilson’s disease is even higher: 150 mg/day for adults and 75 mg/day in 2 to 3 divided doses for children.^14^ The internationally accepted US National Kidney Foundation KDOQI guidelines cover all the major aspects of management of children with CKD. Recommendation 6 (page S53) provides guidance on Dietary Reference Intake (DRI) of vitamin and trace element requirements: “The provision of dietary intake consisting of at least 100% of the DRI for . . . zinc should be considered for children with CKD stages 2 to 5 and 5D.”^15^ In another pediatric study, children on continuous cycling peritoneal dialysis received 8 mg zinc daily (for age >5 years) with no adverse effect but these patients had poor adherence to treatment.^16^ It has been reported that zinc supplementation may reduce copper absorption in the bowel.^17^

### Data From Clinical Routine Visits

*Patient data collected*: It included age, self-reported sex (in all cases equivalent to the sex assignment at birth), etiology of primary kidney disease, date of transplant (if applicable), height, weight, blood pressure, and current medications.
*Baseline blood measurements*: Non-fasting blood samples (~ 5 ml) were obtained at the same time as routine blood tests by outpatient clinic phlebotomists; with an additional 5 mL to measure plasma zinc, serum iFGF-23, cFGF-23, and Klotho. Hemoglobin and serum results of creatinine, electrolytes, urea, bicarbonate, calcium, phosphate, alkaline phosphatase, and parathyroid hormone (PTH) were measured at each institution’s clinical labs. Urine samples collected at the time of blood tests assessed protein, calcium, and phosphate excretion.
*Laboratory tests outside the clinical routine visits*: Plasma zinc was measured by High-Resolution Magnetic Sector Inductively Coupled Plasma Mass Spectrometry (HR-ICP-MS).^18^ Serum 25-hydroxyvitamin D was measured by liquid chromatography and tandem mass spectrometry and accuracy and precision were determined using human serum-based quality controls and standard reference materials (National Institute of Standards and Technology [NIST] Standard Reference Material 972a) as previously described.^19^ Precision values for 25(OH)D3 in our lab are intra-assay CV 6.9%, inter-assay CV 8.2%, and average mean bias −1.3%. Enzyme-linked immunosorbent assay (ELISA) kits measured serum iFGF-23 (Kainos Labs, intra-assay CV 9.7%; inter-assay CV 2.3%), cFGF-23 (Biomedica Immunoassays, intra-assay CV 10.2%; inter-assay CV 22.5%), and human soluble alpha-Klotho (TECO medical, intra-assay CV 12.3%; inter-assay CV 11.2%).

### Sample Size Calculations

Our pilot data indicated that the mean plasma zinc concentration in the cohort of zinc-deficient patients was 9.7 ± 2 µmol/L. The lower reference interval for zinc levels is 11.5 µmol/L. The number of patients required is 2*(1.96+0.84)^2^*2^2^/ (11.5-9.7)^2^ = 20 for treatment group.

### Data Collection, Management, and Analysis

Data were collected at both McMaster and London sites by trained research assistants using a common case report form. REDCap (www.project-redcap.org) software was used to capture and store de-identified study data and then transferred to Excel spreadsheets for further analysis. Statistical analysis was done using Student’s *t*-test, Mann-Whitney test, Wilcoxon matched pairs signed-rank test, linear and multivariable regression analysis, as appropriate after assessing normality. Normally distributed data were expressed as mean ± 1 standard deviation, otherwise median and interquartile range were cited. *P* value of <.05 was considered statistically significant.

## Results

Participant demographic, anthropometric, disease stage, medication, and laboratory characteristics are summarized in Table 1. Zinc deficiency (defined as a plasma concentration at baseline <11.5 µmol/L) was found in 22 patients (53.7%).

**Table 1.** Participant Baseline Demographical and Biochemical Characteristics (N = 41).

Parameter	Mean (SD)	Median [IQR]
Age	12.9 (4.13)	14.4 [9.20, 16.10]
Male, No (%)	25 (61.0)	
Height (cm)	147.06 (22.40)	154.00 [131.90, 163.90]
Weight (kg)	47.47 (19.83)	51.00 [29.70, 60.30]
CKD, No (%)		
Stage 2	17 (41.5)	
Stage 3	15 (36.6)	
Stage 4	9 (22.0)	
Taking ACE inhibitors, N (%)	13 (31.7)	
Taking vitamin D supplement, N (%)	23 (56.1)	
Taking calcitriol, N (%)	11 (26.8)	
Serum calcium (mmol/L)	2.39 (0.12)	2.39 [2.31, 2.48]
Serum phosphate (mmol/L)	1.27 (0.27)	1.21 [1.12, 1.36]
Serum alkaline phosphate (mmol/L)	174.30 (100.86)	155.00 [85.00, 240.00]
Serum PTH (pmol/L)	6.29 (4.98)	5.35 [2.90, 7.85]
Serum 25 OHD nmol/L	69.40 (25.33)	66.70 [58.20, 82.50]
Serum calcitriol (1,25 OHD) pmol/L	76.15 (49.53)	75.00 [36.00, 119.00]
Kidney PO_4_ reabsorption (TMP/GFR)	1.05 (0.30)	1.04 [0.88, 1.18]
Urine Ca/Creatinine ratio (mmol/mmol)	0.18 (0.16)	0.10 [0.06, 0.32]
Fractional excretion of phosphate	18.67 (13.02)	16.85 [9.43, 25.40]
Plasma zinc (µmol/L)	11.34 (2.05)	11.30 [10.30, 12.50]
Serum Intact FGF-23 (pg/ml)	73.88 (51.68)	56.00 [38.75, 92.15]
Serum C-terminal FGF-23 (pmol/L)	2.67 (2.13)	1.89 [1.11, 3.39]
Serum Klotho (pg/mL)	1036.74 (657.77)	793.48 [592.43, 1284.08]
Hemoglobin (g/L)	122.95 (13.56)	121.00 [117.00, 131.00]

*Note.* eGFR = estimated glomerular filtration rate; ACE = angiotensin-converting enzyme; PTH = parathyroid hormone; 25 OHD = 25-hydroxy vitamin D; PO_4_ = phosphate; Ca = calcium; Zn = zinc; FGF-23 = fibroblast growth factor 23.

**Figure 1.**
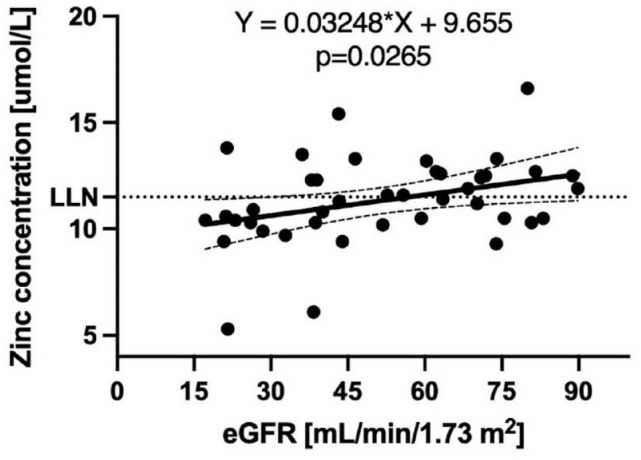
The relationship between eGFR and serum zinc concentration at study entry in the 41 patients. The equation of the linear regression line is also provided, and the relationship was significant.

Table 2 provides baseline participant characteristics stratified by zinc status. No differences in characteristics were observed between subgroups of patients with CKD with native and transplanted kidneys. A linear regression model exploring relationship between zinc level and eGFR suggested that for every 1 unit drop of eGFR, zinc concentration dropped by 0.026 µmol/L (*P* = .04). The relationship between eGFR and zinc levels is depicted in Figure 1. A multivariate regression model exploring relationships between of eGFR, zinc status, and intact FGF-23 revealed that the *P* value for the latter becomes insignificant and reduction in eGFR plays the key role in reduction of zinc levels.

**Table 2.** Patient Baseline Characteristics Stratified by Zinc Status.

Parameter	Sufficient (n = 19)	Deficient^a^ (n = 22)
Age	12.12 (4.36)	13.65 (3.88)
Male, N (%)	13 (68.4)	12 (54.5)
Height (cm)	142.29 (23.96)	151.19 (20.63)
Weight (kg)	43.88 (19.78)	50.57 (19.81)
eGFR (mL/min/1.73 m^2^)	60.17 (19.23)	44.54 (21.73)
CKD, N (%)		
Stage 2	11 (57.9)	6 (27.3)
Stage 3	7 (36.8)	8 (36.4)
Stage 4	1 (5.3)	8 (36.4)
Taking ACE inhibitors N (%)	5 (26%)	8 (36%)
Taking Vitamin D suppl, N (%)	13 (68.4)	10 (45.5)
Taking calcitriol, N (%)	4 (21.1)	7 (31.8)
Calcium (mmol/L)	2.44 (0.11)	2.34 (0.11)
Phosphate (mmol/L)	1.29 (0.23)	1.25 (0.30)
Alkaline phosphate (mmol/L)	195.00 (112.27)	152.44 (84.93)
PTH (pmol/L)	3.97 (2.61)	8.39 (5.69)
Serum 25 OHD (nmol/L)	77.24 (26.69)	63.34 (23.02)
Calcitriol (pmol/L)	74.62 (63.29)	77.59 (33.87)
c-Terminal FGF-23 (pmol/L)	2.12 (1.94)	3.11 (2.22)
Intact FGF-23 (pg/mL)	53.71 (30.06)	91.17 (60.22)
Klotho (pg/ml)	1205.44 (799.46)	891.05 (477.80)
Copper (µmol/L)	17.37 (3.82)	17.88 (7.53)
Hemoglobin (g/L)	127.00 (10.41)	119.45 (15.15)
TmP-GFR	1.14 (0.21)	0.96 (0.36)
Urine Ca/Creatinine (mmol/mmol)	0.20 (0.18)	0.16 (0.15)

*Note.* eGFR = estimated glomerular filtration rate; ACE = angiotensin-converting enzyme; PTH = parathyroid hormone; 25-OHD = 25-hydroxy vitamin D; PO_4_ = phosphate; Ca = calcium; Zn = zinc; FGF-23 = fibroblast growth factor 23; TmP/GFR = maximum tubular phosphate reabsorption in mass per unit volume of glomerular filtration rate.

a Deficiency defined as serum 25-OHD <50 nmol/L.

We identified 22/41 (54%) patients with native or transplant CKD who were zinc deficient. Of those, 14 patients (9 with CKD in native kidneys and 5 with CKD in transplant kidneys) (64% enrollment rate) who had provided consent and were successfully followed up, received zinc supplements for 3 months and normalization of plasma zinc concentration was achieved in 8 patients (5 with CKD with CKD in native kidneys and 3 with CKD in transplant kidneys (57%)). Mean plasma zinc concentration rose significantly (*P* = .0038) from 10.04 ± 1.42 to 12.29 ± 3.77 μmol/L after 3 months of zinc treatment.

No side effects, nor any adverse events were reported by the participants and no reduction in plasma copper was observed in any participant as pre-treatment and post-treatment concentrations were similar at 19.9 ± 8.6 µmol/L and 19.3 ± 8.5 µmol/L, respectively (*P* = .93).

There were no statistically significant changes in bone health biomarkers following zinc treatment. Intact FGF-23, cFGF-23, Klotho levels, and phosphate excretion remained the same (Table 3).

**Table 3.** Comparison of Pre-intervention and Post-intervention Measurements of Biomarkers in Patients Treated With Zinc Citrate.

Biomarker, mean (SD)	First measurement	Second measurement	*P*-value
Zinc level (µmol/L)			
Overall	10.04 (1.42)	12.29 (3.77)	.004
CKD in native kidneys (n = 9)	9.77(1.67)	11.32 (2.79)	.01
CKD in kidney transplant (n = 5)	10.5 (0.78)	14.24 (5.01)	.07
Creatinine (µmol/L)	151.00 (64.16)	163.75 (96.83)	.37
eGFR (mL/min/1.73 m^2^)	44.32 (22.01)	44.99 (24.67)	.67
Calcium (mmol/L)	2.37 (0.09)	2.36 (0.17)	.85
Phosphate (mmol/L)	1.30 (0.34)	1.40 (0.59)	.60
Alkaline phosphate (mmol/L)	151.36 (92.35)	162.43 (112.55)	.42
Parathyroid hormone (pmol/L)	7.14 (3.82)	9.65 (5.38)	.12
Calcitriol (pmol/L)	70.43 (31.20)	83.20 (44.30)	.13
Hemoglobin (g/L)	118.00 (12.31)	121.81 (13.28)	.08
iFGF-23 (pg/mL)	84.99 (64.71)	106.02 (91.87)	.22
cFGF-23 (pmol/L)	2.80 (2.40)	3.15 (2.72)	.60
Klotho (pg/ml)	853.86 (501.89)	987.61 (652.03)	.34
25-OHD (nmol/L)	65.75 (24.62)	73.27 (21.11)	.64
Copper (µmol/L)	18.39 (8.74)	18.31 (8.16)	.30
Urine calcium (mmol/L)	0.91 (1.27)	0.84 (1.12)	.73
Urine phosphate (mmol/L)	9.14 (6.57)	12.20 (10.80)	.44
Urine creatinine (mmol/L)	6.24 (4.16)	5.95 (3.91)	.60
Urine Ca/Creatinine (mmol/mmol)	0.16 (0.14)	0.16 (0.14)	1
TmP-GFR	0.98 (0.39)	1.08 (0.47)	.50
Fractional excretion of phosphate	24.05 (16.22)	23.45 (15.24)	.90

*Note.* iFGF-23 = intact fibroblast growth factor 23; cFGF-23 = c-terminal fibroblast growth factor 23; OHD = hydroxy vitamin D; TmP/GFR = maximum tubular phosphate reabsorption in mass per unit volume of glomerular filtration rate.

Further review of data showed impact of zinc therapy on cFGF-23 if 3 patients taking calcitriol were excluded from biomarker analysis due to its significant impact on FGF-23 and Klotho metabolism (Table 4).

**Table 4.** Selected Biomarkers Change in Response to Zinc Supplementation in Study Participants With CKD not Treated With Calcitriol (n = 11).

Biomarker	First measurement	Second measurement	*P*-value
eGFR, mean	53.1 (21.3)	54.2 (23.8)	.51
Zinc (μmol/L)	10.3 (9.8-10.9)	11.8 (10.8-13.18)	.03*
iFGF-23 (pg/mL)	60.7 (44.95-89.5)	64.6 (53.7-142.9)	.08
cFGF-23 (pmol/L)	2.15 (1.60)	3.42 (2.72)	.008*
Klotho (pg/mL)	826.26 (445.31)	812.14 (501.63)	.37
25-OHD (nmol/L)	61.1 (55.9-74.2)	67.5 (63.8-76.1)	.43
TmP-GFR	0.97 (0.39)	1.04 (0.25)	.96

*Note.* iFGF-23 = intact fibroblast growth factor 23; cFGF-23 = c-terminal fibroblast growth factor 23; OHD = hydroxy vitamin D; TmP/GFR = maximum tubular phosphate reabsorption in mass per unit volume of glomerular filtration rate.

* highlights statistically significant results.

## Discussion

Zinc is a part of more than 300 enzymes in the human body and plays a role in gene expression and is essential for cell division and development of multiple organs including the kidneys. Unfortunately, it is not well studied in CKD and a PubMed search revealed only 1 study in the last 10 years that studied the effect of zinc supplementation in children with CKD.^20^

Our study examined the relationships between plasma zinc, kidney function, and biomarkers of metabolic bone disease (25(OH)D, iFGF-23, cFGF-23, and Klotho) in children with native and transplant-associated CKD. Children with lower GFR were frequently zinc deficient and plasma zinc progressively fell with a reduction in eGFR. Zinc deficiency was associated with higher plasma/serum iFGF-23; however, iFGF-23 concentration was predominantly determined by the falling GFR. Glomerular filtration rate appears to be the most important factor responsible for FGF-23 elevation.^4^ Zinc deficient and sufficient children had similar circulating cFGF-23 and alpha-Klotho. Zinc supplementation for 3 months restored plasma zinc to normal in about a 50% of the zinc-deficient patients with CKD. A longer course of therapy and a higher dose may be required in patients who did not achieve normal plasma zinc, but this was beyond the scope of our study. In children treated with zinc but not receiving calcitriol a statistically significant rise in cFGF-23 was observed. However, there were no other meaningful changes to biochemical measures or an increase in phosphaturia. Unlike the data in mice,^9^ there was no rescue effect of zinc supplementation on Klotho in children with primary CKD or CKD due to transplant failure. Zinc supplementation was tolerated and appears to be safe in these children as there were no reported side effects and plasma copper remained unchanged.

Chronic kidney disease–associated metabolic bone disease is one of the major challenges for patients with CKD, both before^21^ and after kidney transplantation.^22^ Worsening kidney function leads to reduced excretion of phosphate and its accumulation in the body. Reduction of phosphate is the cornerstone of treatment in these patients and can be achieved by restricting its consumption in combination with oral phosphate binders. Both measures are often unsuccessful due to difficulties with diet adherence and phosphate binder side effects.^23^ FGF-23 is a phosphaturic hormone that stimulates the kidneys to increase phosphate removal and decrease production of 1,25-dihydroxyvitamin D, the active hormone.^24^ In order to exert its function, FGF-23 requires the presence of the Klotho protein which circulates in blood and is also expressed by the tubular cells^25^ and the parathyroid gland.^5^ Patients with CKD have very low levels of Klotho,^26^ and as a result, despite having high levels of FGF-23, they are unable to remove excess phosphate due to Klotho deficiency as well as the ability of FGF-23 to suppress PTH at the parathyroid gland owing to reduced expression of PTH in CKD.^5^ Limited data exist on strategies to upregulate Klotho production and expression on the parathyroid gland. It is thought that inhibitors of angiotensin- converting enzyme may play a role, as Klotho is suppressed by angiotensin, but no studies to explore this hypothesis have been reported. Our study followed on the animal models which suggested that zinc supplementation can increase circulating Klotho.^9^ In this exploratory study, improvement in zinc status did not alter circulating Klotho nor reduce phosphaturia. Future studies should be powered to address potential role of zinc on Klotho in modulating FGF-23 and Klotho functions as there is the potential to improve cardiovascular morbidity and mortality in patients with CKD.

Many knowledge gaps exist regarding the role of zinc in health and CKD even though it is a constituent of more than 300 enzymes in the human body, plays a role in gene expression, and is essential for cell division/development of multiple organs including the kidneys. Whereas selenium deficiency in adults is more prevalent, zinc deficiency is frequently observed in pediatric CKD patients.^2^ The beneficial effect of zinc on the immune system and its direct gastrointestinal effect have led to oral zinc therapy in the prevention and treatment of diarrhea in children. Other consequences of zinc deficiency are anorexia,^27^ growth retardation,^28^ and disorders of neurodevelopment.^29^ Most of these disorders are seen in patients with CKD, and it is possible that zinc deficiency may worsen them, but few studies have explored it. Zinc deficiency has also been shown to aggravate blood pressure in hypertension, tubulointerstitial nephropathy, glomerular hemodynamics, and progression of renal failure.^30^ Zinc sufficiency plays an important role in preservation of kidney function in rats.^31^ In experimental models, severe zinc deficiency induced a reduction of GFR and kidney blood flow and increased renal vascular resistance. In clinical studies, kidney disease progression in zinc deficient and sufficient patients has yet to be compared, but data have shown a high prevalence of zinc deficiency in CKD patients who were under either conservative management or dialysis.^32^ This is attributed to reduced nutritional intake of zinc in CKD patients due to poor appetite secondary to uremia and/or to a redistribution of zinc in the body with increased accumulation of zinc in the liver due to an unknown mechanism. Patients with kidney transplants display similar zinc deficiency.^33^ To date, there are no clinical guidelines regarding the dose, duration, or effectiveness of zinc therapy in zinc-deficient children with CKD, and further research is required. However, our study has begun to address some of these knowledge gaps. From our exploratory study, zinc supplementation at described doses can be recommended as safe in CKD children, but supplementation and monitoring for longer than 3 months may be required to achieve normal zinc status.

### Limitations

Our study involved a small number of patients, but which met the estimate of our sample size calculation. Even though this was a joint study of 2 tertiary pediatric centers we had to combine patients with native and transplant-associated CKD. One might argue that these groups should be separate due to subtle differences in calcium/phosphate metabolism. We also did not monitor adherence and dietary zinc intake. These limitations potentially reduce the generalizability.

## Conclusions

This was an exploratory study from which the data can be used to design a larger clinical trial to further define any benefits derived from zinc supplementation in the CKD population. Overall, it appears that zinc status is related to kidney function and possibly connected to bone metabolism in patients with CKD. However, it plays a minor role in fine-tuning various metabolic processes and therapeutic zinc supplementation seems to improve bone metabolism only modestly in asymptomatic CKD patients. In our cohort, 3-month supplementation of oral zinc seemed to be enough to replete zinc levels only in half of deficient patients.
